# Biodegradation of two organophosphorus pesticides in whole corn silage as affected by the cultured *Lactobacillus plantarum*

**DOI:** 10.1007/s13205-016-0364-3

**Published:** 2016-02-16

**Authors:** Ying-Hua Zhang, Di Xu, Xin-Huai Zhao, Yao Song, Yan-Le Liu, Hong-Nan Li

**Affiliations:** 1 Key Laboratory of Dairy Science, Ministry of Education, Northeast Agricultural University, Harbin, 150030 People’s Republic of China; 2 Department of Food Science, Northeast Agricultural University, Harbin, 150030 People’s Republic of China; 3 Synergetic Innovation Center of Food Safety and Nutrition, Northeast Agricultural University, Harbin, 150030 People’s Republic of China

**Keywords:** Corn silage, Chlorpyrifos, Phorate, Degradation, *Lactobacillus plantarum*

## Abstract

Biodegradation of the pesticides is considered as one of the safest and cheapest ways. The aim of the present study was to reveal if the inoculated *Lactic acid bacteria* widely used in silage could provide beneficial safety to guarantee dairy feedstuffs and fresh milk. Organophosphorus pesticides (OPPs) degradation in whole corn silage was investigated. Two OPPs, chlorpyrifos and phorate, were added to the whole corn, which was inoculated with *L*. *plantarum* 1.0315, *L*. *plantarum* 1.0624, *L*. *plantarum* 1.0622 and their combination at room temperature for 10 weeks. OPPs residues in the whole corn silage at different weeks were analyzed by gas chromatography after OPPs extraction and purification. The degradation rate constants were calculated according to the OPPs residues results at different fermentation stage. The data showed that the inoculated microorganisms and strain combination exhibited obvious acceleration on OPPs degradation as the wild microorganisms did, and resulted in decreased levels of OPPs from 24.9 to 33.4 %. Strains combination brought about greater OPPs degradation than single strain or the wild microorganisms. Compared to phorate, clorpyrifos had lower degradation rate constants (0.0274–0.0381 vs. 0.0295–0.0355 week^−1^) and was more stable. The present result indicates safety benefit of *lactic acid bacteria* on corn silage besides lactic acid fermentation.

## Introduction

Organophosphorus pesticides (OPPs) are a group of toxic agricultural chemicals widely used to control insect pests, plant pathogens, and weeds for plant protection to increase agricultural productivity. OPPs bring about enormous economic benefits to the farmers, and also raise potential risk to the health of animal especially humans (Sogorb et al. 2004). OPPs will pollute the environment eventually reach water (Na et al. 2006), soil (Cycón et al. 2009; Singh et al. 2004), animal feeds (Kumar et al. 2013). For example, OPPs were detected in the corn planted in Ghana (Akoto et al. 2013). The remaining OPPs in silage might not lead to the critical contamination of fresh milk and dairy products, but some analyses did observe OPPs residues in milk and dairy products (Tsiplakou et al. 2010; Battu et al. 2004). OPPs pollution in processed foods has been paid more and more attention all over the world (Abdel-Halim et al. 2006). There were some studies using microbial degradation to control OPPs (El Fantroussi and Agathos 2005; Fang et al. 2006), as biodegradation of the pesticides is considered as one of the safest and cheapest ways.

*Lactic acid bacteria* (LAB) as the natural intestinal microbiota of most animals (Rojo-Bezares et al. 2006), is one of the most common microorganisms in foods (De Vuyst and Leroy 2007), and also widely used in silage and inoculated in an effort to improve their preservation or quality (Addah et al. 2010; Danner et al. 2003; Filya 2003). Some intestinal microorganisms including LAB were efficient in degrading the OPPs (Harishankar et al. 2013). Two researchers reported that LAB had ability to enhance the degradation of some OPPs in the skimmed milk (Bo et al. 2011; Zhao and Wang 2012). Islam et al. (2010) revealed the OPPs biodegradation mechanism by expressing a gene encoding OPPs hydrolase enzyme from LAB. The phosphatase produced by LAB in the fermentation system might be the important factor for OPPs degradation (Zhang et al. 2014). *L*. *plantarum* as the main inoculants in the silage processing has the ability to accelerate OPPs degradation. Unfortunately, there were few researches studying OPPs degradation in silage. In the present study, OPPs degradation in whole corn silage was investigated. The potential impacts of three LAB including *L*. *plantarum* 1.0622, *L*. *plantarum* 1.0315 and *L*. *plantarum* 1.0624 on the degradation of chlorpyrifos and phorate were evaluated and compared, based on the calculated degradation kinetics of first-order reaction. The aim of the present study was to reveal if the inoculated LAB could provide beneficial safety guarantee to dairy feedstuffs and fresh milk.

## Materials and methods

### Materials

Chlorpyrifos and phorate were purchased from Sigma Chemical Co. (Saint Louis, MO, USA.), with declared purity from 94.5 to 99.5 %, respectively, and stored at 4 °C before use.

Three *lactobacillus plantarum* named *L*. *plantarum* 1.0315, *L*. *plantarum* 1.0624 and *L*. *plantarum* 1.0622 were obtained from the Centre of Lactic Acid Bacteria in Key Laboratory of Dairy Science, Ministry of Education, Northeast Agricultural, University (Harbin, China). The chemicals and solvents used were analytical and chromatographic agents. Water used was highly purified water prepared with Milli-Q PLUS (Millipore Corporation, New York, NY, USA).

Whole corn from Xiangfang farm near Harbin was harvested on 20 September, 2013, and chopped into a length of 1–2 cm by a conventional forage harvester under farm conditions.

### Silage preparation

The following treatments were applied to whole corn to prepare three groups: (1) control groups (containing wild microorganisms), whole corn without any further treatment; (2) reference group (containing inactivated wild microorganisms), whole corn heated at 115 °C for 15 min to inactivate the wild microorganisms; (3) treated groups, whole corn subjected to heating inactivation and LAB inoculation. After the treatments, the whole corn in each group was spiked with chlorpyrifos and phorate at about 0.36 mg/kg by a hand sprayer, manually mixed well. After then, the whole corn in treated group was inoculated with the selected strains and combination, respectively, by a level of 1 × 10^6^ cfu/g. All prepared samples were sealed in plastic bags and stored at room temperature (about 20 °C) for 10 weeks. During the storage, the samples in each group were assayed for the OPPs residues every week.

### Sample extraction and purification

Silage samples of 2.0 g and activated carbon of 0.2 g were mixed and extracted with 10 mL dichloromethane by a shaking for 10 min. The liquid phase was decanted to a separatory funnel, and the residues left were re-extracted twice with 10 mL dichloromethane. The collected liquid phase was joined and dried through anhydrous sodium sulphate (about 3 g) for 20 min. The dichloromethane phase of 5 mL was evaporated to dry at 30 °C by blowing nitrogen gas. The residues were reconstituted into 1.0 mL by acetone and filtered through a 0.45 μm microporous membrane before GC analysis.

### GC analysis of organophosphorus pesticides

OPPs in the samples were quantified by an Agilent 7890 Gas Chromatography (Agilent Technologies, Inc, Santa Clara, CA, USA) with a capillary column (DB-1701, 30 m × 0.25 mm × 0.25 mm) and a flame photometric detector. Flow rate of the carrier gas (nitrogen gas) was 3 mL/min. The temperatures of injection, column and detector were set at 200, 90 and 250 °C, respectively. The purified sample (1.0 μL) was detected under programmed temperature gradient from initial temperature of 90 °C for 1 min, heating from 100 to 260 °C at 10 °C/min, holding for 3 min at 260 °C. Quantification of the pesticides was carried out by comparison of their peak areas with a calibrated standard curve with multitude-point calibration.

The relative decreasing levels of OPPs was calculated as the decreased concentrations of OPPs at the last week compared with the beginning ones.

### Statistical analyses

All data were expressed as mean ± SD (standard deviations) from three independent trials. Kinetic parameters were calculated with linear regression analysis. SPSS 16.0 software (SPSS Inc., Chicago, IL, USA) was used to analyze the data.

## Results and discussion

### GC analysis of OPPs in corn silage

**Fig. 1:**
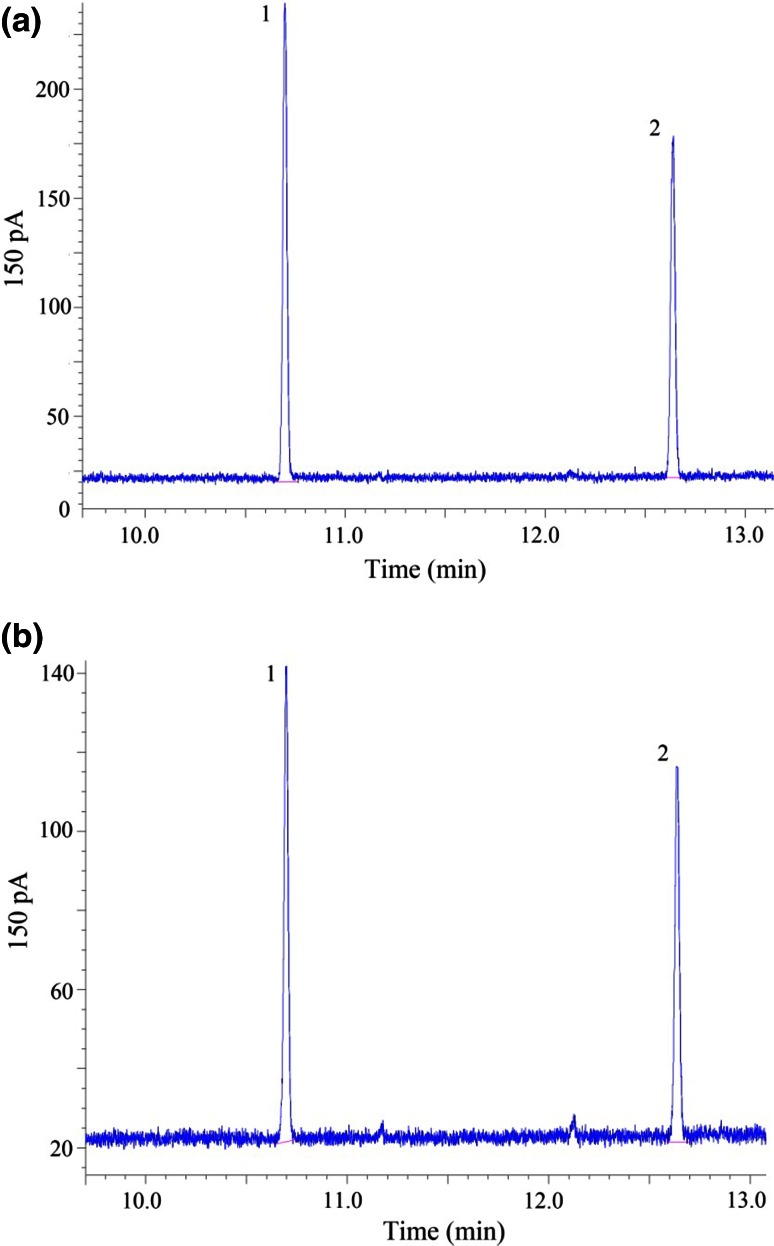
Typical GC profiles of two OPPs for a standard solution (**a**) and a corn silage sample (**b**), respectively. Peak 1 to 2 represent phorate, chlorpyrifos, respectively

A liquid–liquid extraction method is suitable method to extract OPPs thoroughly (Ballesteros and Parrado 2004), as the present study did. Typical GC profiles of the OPPs for a sample were given in Fig. 1, which showed both chlorpyrifos and phorate were well-separated. Detection limits of chlorpyrifos and phorate were 0.007 and 0.006 mg/kg, ensuring a precise measurement in the corn silage. The recoveries were 84.1–113.1 % (at 0.2–0.8 mg/kg spiking levels), with the standard deviations of 3.3 and 2.9 %, respectively (Table 1).

**Table 1:** Recoveries and detection limits of chlorpyrifos and phorate in corn silage

Pesticides	Recoveries at three spiking levels (mg/kg)			Standard deviations (%)	Detection limits (mg/kg)
	0.20	0.40	0.80		
Chlorpyrifos	85.5	85.8	84.1	3.3	0.007
Phorate	90.9	105.6	113.1	2.9	0.006

### OPPs dissipation in corn silage

In the present study, three strains (*L*. *plantarum*) and their combinations were inoculated in fresh whole corn silage. The silage was then fermented at ambient temperature for 10 weeks. The assayed results indicated that OPPs had dissipated (Table 2), as the detected OPPs concentration in all samples showed a decrease trends. *L*. *plantarum* and their combinations inoculated in whole corn of five treated groups showed significant acceleration on OPPs dissipation, comparing to the OPPs dissipation in whole corn of reference group (with microorganism inactivation). After storage of 10 weeks, the decreasing levels for chlorpyrifos and phorate in the control group were 27.6 and 26.2 %, while those levels for chlorpyrifos and phorate in the reference group were only 7.3 and 7.5 %, respectively. The reference group was whole corn heated at 115 °C for 15 min to inactivate the microorganisms completely in order to avoid the effect of the wild microorganism. Promoting OPPs degradation by inoculated LAB in the treated groups was determined by comparing with the control. At the same time, the decreasing levels for chlorpyrifos and phorate in the treated groups ranged from 24.9 (phorate, incubated by *L*. *plantarum* 1.0624) to 33.4 % (phorate, incubated by *L*. *plantarum* 1.0622 and *L*. *plantarum* 1.0315). It thus was seen that the inoculated microorganisms also brought about OPPs dissipation, and the applied strain combinations had much stronger acceleration of OPPs dissipation than single strains and wild microorganisms. Unfortunately, single strain applied in the samples of treated groups resulted in similar OPPs dissipation as the wild microorganisms did in the samples of control groups. Application strain combination in the whole corn silage was thus suggested to grant much OPPs dissipation.

**Table 2:** Measured concentration and degradation rate constants of chlorpyrifos and phorate in corn silage during storage

Pesticides	Storage times (week)	The measured concentrations (mg/kg) in three groups^a^						
		Control	Reference	Treated-A	Treated-B	Treated-C	Treated-D	Treated-E
Chlorpyrifos	0	0.359 ± 0.002	0.342 ± 0.004	0.363 ± 0.009	0.355 ± 0.002	0.354 ± 0.005	0.358 ± 0.003	0.360 ± 0.008
	1	0.347 ± 0.009	0.339 ± 0.007	0.344 ± 0.010	0.346 ± 0.004	0.341 ± 0.007	0.345 ± 0.008	0.346 ± 0.003
	2	0.337 ± 0.007	0.338 ± 0.006	0.325 ± 0.003	0.331 ± 0.006	0.326 ± 0.005	0.325 ± 0.003	0.326 ± 0.002
	3	0.325 ± 0.006	0.335 ± 0.003	0.312 ± 0.003	0.313 ± 0.006	0.311 ± 0.002	0.309 ± 0.002	0.319 ± 0.003
	4	0.311 ± 0.004	0.333 ± 0.005	0.299 ± 0.009	0.301 ± 0.005	0.299 ± 0.005	0.301 ± 0.003	0.308 ± 0.006
	5	0.303 ± 0.009	0.330 ± 0.005	0.290 ± 0.003	0.286 ± 0.004	0.290 ± 0.003	0.291 ± 0.004	0.298 ± 0.006
	6	0.295 ± 0.006	0.326 ± 0.005	0.271 ± 0.007	0.278 ± 0.002	0.286 ± 0.004	0.285 ± 0.001	0.287 ± 0.004
	7	0.286 ± 0.008	0.323 ± 0.004	0.268 ± 0.005	0.275 ± 0.001	0.276 ± 0.003	0.277 ± 0.001	0.280 ± 0.004
	8	0.281 ± 0.009	0.321 ± 0.003	0.265 ± 0.004	0.270 ± 0.002	0.273 ± 0.003	0.273 ± 0.001	0.272 ± 0.001
	9	0.266 ± 0.004	0.320 ± 0.004	0.260 ± 0.001	0.266 ± 0.002	0.268 ± 0.002	0.262 ± 0.001	0.263 ± 0.002
	10	0.260 ± 0.003	0.317 ± 0.005	0.255 ± 0.006	0.264 ± 0.002	0.264 ± 0.001	0.257 ± 0.001	0.259 ± 0.001
Phorate	0	0.343 ± 0.003	0.320 ± 0.009	0.357 ± 0.009	0.348 ± 0.001	0.342 ± 0.003	0.349 ± 0.003	0.368 ± 0.002
	1	0.335 ± 0.002	0.318 ± 0.010	0.325 ± 0.007	0.328 ± 0.007	0.328 ± 0.007	0.339 ± 0.003	0.345 ± 0.007
	2	0.319 ± 0.008	0.316 ± 0.011	0.309 ± 0.007	0.308 ± 0.005	0.306 ± 0.009	0.318 ± 0.008	0.316 ± 0.004
	3	0.317 ± 0.006	0.315 ± 0.011	0.297 ± 0.002	0.304 ± 0.007	0.305 ± 0.001	0.317 ± 0.006	0.310 ± 0.012
	4	0.311 ± 0.004	0.312 ± 0.009	0.288 ± 0.001	0.297 ± 0.008	0.296 ± 0.007	0.303 ± 0.007	0.300 ± 0.005
	5	0.296 ± 0.010	0.310 ± 0.006	0.279 ± 0.004	0.283 ± 0.002	0.285 ± 0.009	0.291 ± 0.011	0.288 ± 0.007
	6	0.294 ± 0.001	0.306 ± 0.003	0.275 ± 0.003	0.279 ± 0.004	0.282 ± 0.003	0.290 ± 0.008	0.284 ± 0.002
	7	0.288 ± 0.004	0.303 ± 0.002	0.269 ± 0.003	0.275 ± 0.005	0.276 ± 0.002	0.275 ± 0.011	0.274 ± 0.006
	8	0.264 ± 0.001	0.301 ± 0.006	0.263 ± 0.003	0.267 ± 0.009	0.264 ± 0.004	0.263 ± 0.010	0.256 ± 0.008
	9	0.263 ± 0.002	0.299 ± 0.005	0.255 ± 0.004	0.262 ± 0.004	0.262 ± 0.003	0.262 ± 0.003	0.254 ± 0.007
	10	0.253 ± 0.001	0.296 ± 0.008	0.247 ± 0.004	0.256 ± 0.001	0.257 ± 0.004	0.254 ± 0.003	0.245 ± 0.006

^a^The symbols A–E in treated groups represent the stains used. A, *L*. *plantarum* 1.0622; B, *L*. *plantarum* 1.0315; C, *L*. *plantarum* 1.0624; D, *L*. *plantarum* 1.0315 and *L*. *plantarum* 1.0624; E, *L*. *plantarum* 1.0622 and *L*. *plantarum* 1.0315

*L*. *plantarum* was able to degrade OPPs in the milk (Bo et al. 2011; Zhao and Wang 2012). The present result shared the same conclusion to these researches. Other LAB are capable of being applied in silage, their acceleration on OPPs dissipation needs a detailed investigation in future.

### Degradation kinetics of OPPs

Degradation rate constants and half live periods of chlorpyrifos and phorate were calculated from the data in Table 2, based on a reported first-order reaction model (Vanclooster et al. 2000). The calculated results of degradation kinetics were listed in Table 3. The degradation of chlorpyrifos and phorate in whole corn during storage of 10 weeks fitted well to first-order reaction model, reflected by the regression coefficient *R* ^*2*^ (0.945–0.996 for chlorpyrifos and 0.945–0.989 for phorate). Chlorpyrifos was more stable than phorate. Chlorpyrifos had lower rate constant (0.0295–0.0355 week^−1^), while phorate showed higher one (0.0274–0.0381 week^−1^). Further, *L*. *plantarum* combinations led to stronger OPPs degradation than the single strain only except *L*. *plantarum* 1.0622 for chlorpyrifos. The rate constants resulted by *L*. *plantarum* combinations were 0.0320–0.0381 week^−1^, while the rate constants resulted by single strain and wild microorganisms were 0.0274–0.0355 and 0.0300–0.0321 week^−1^, respectively.

**Table 3:** Degradation kinetics of chlorpyrifos and phorate in corn silage cultured with lactic acid bacteria at room temperature

Pesticides	Sample groups and the inoculated strains^a^	Parameters		
		*k* (week^−1^)	*R* ^2^	*t*_1/2_ (week)
Chlorpyrifos	Control	0.0321	0.996	21.6
	Reference	0.0078	0.991	88.9
	Treated-A	0.0355	0.957	19.5
	Treated-B	0.0317	0.945	21.9
	Treated-C	0.0295	0.961	23.5
	Treated-D	0.0323	0.974	21.5
	Treated-E	0.0329	0.990	21.1
Phorate	Control	0.0300	0.972	23.1
	Reference	0.0080	0.989	86.6
	Treated-A	0.0322	0.945	21.5
	Treated-B	0.0284	0.961	24.4
	Treated-C	0.0274	0.969	25.3
	Treated-D	0.0320	0.985	21.7
	Treated-E	0.0381	0.972	18.2

*k* degradation rate constant, *R* ^*2*^ regression coefficient, *t*_1/2_ half life period

^a^The symbols A–E in treated groups represent the stains used. A, *L*. *plantarum* 1.0622; B, *L*. *plantarum* 1.0315; C, *L*. *plantarum* 1.0624; D, *L*. *plantarum* 1.0315 and *L*. *plantarum* 1.0624; E, *L*. *plantarum* 1.0622 and *L*. *plantarum* 1.0315

OPPs degradation kinetics in milk by LAB had been investigated, indicating LAB was helpful to OPPs degradation (Bo et al. 2011; Zhao and Wang 2012). In a study of Sharma et al. (2005), degradation of some pesticides in wheat flour was also studied. Cho et al. (2009) found that chlorpyrifos could be degraded rapidly by LAB during the fermentation of Kimchi. These mentioned results were consistent with the present result. Unfortunately, the rate constants of chlorpyrifos and phorate in whole corn of control and treated groups were very low (about 0.0274–0.0381 week^−1^, Table 3). These values were equal to 1.63–2.27 × 10^−4^ h^−1^. On the contrary, the reported rate constants of some OPPs in milk were 0.0153–0.0420 h^−1^ (Zhao and Wang 2012). Three factors were considered here to be responsible for slow OPPs degradation in the whole corn. Firstly, the whole corn was kept in a lower temperature than the cultured milk, which might bring about lower OPPs degradation. Secondly, the whole corn was in solid state, which retarded the transportation of OPPs to the inoculated microorganisms. Lastly, the whole corn had much lower water activity than the milk, which might also be favor to the stability of the studied OPPs.

In conclusion, the inoculated *L*. *plantarum* strain and its combination, as well as wild microorganisms in planted corn all had accelerating effect on chlorpyrifos and phorate degradation during the storage, which led to greater dissipation of chlorpyrifos and phorate in the corn silage. Chlorpyrifos was more stable than phorate. Strain combinations totally showed greater acceleration on the degradation of chlorpyrifos and phorate, and thus should be more potential to practical application. Inoculation of corn silage by *L*. *plantarum* could provide safety benefit to corn silage by reducing the risk of OPPs pollution.
